# Delay in seeking treatment before emergent heart failure readmission and its association with clinical phenotype

**DOI:** 10.1186/s40560-020-00482-z

**Published:** 2020-08-26

**Authors:** Makoto Takei, Kazumasa Harada, Yasuyuki Shiraishi, Junya Matsuda, Yoichi Iwasaki, Yoshiya Yamamoto, Kenichi Matsushita, Tetsuro Miyazaki, Takamichi Miyamoto, Kiyosi Iida, Shuzo Tanimoto, Yuji Nagatomo, Toru Hosoda, Shun Kohsaka, Takeshi Yamamoto, Ken Nagao, Morimasa Takayama

**Affiliations:** 1 Tokyo CCU Network Scientific Committee, Tokyo, Japan; 2 Department of Cardiology, Saiseikai Central Hospital, Mita 1-4-17, Minato-ku, Tokyo, 108-0073 Japan

**Keywords:** Heart failure, Emergency medical service, Delay in seeking treatment, Phenotype

## Abstract

**Background:**

Many patients with emergent heart failure (HF) readmission have a delay between symptom onset and hospitalization. The present study aimed to characterize the interval between symptom onset and hospitalization in patients being readmitted for HF and to compare the clinical phenotypes of patients with delay before emergent readmission with those who presented to the hospital earlier.

**Methods:**

Data for a total of 2073 consecutive patients was collected from the Tokyo CCU Network database; the patients were divided into delayed (those who sought medical help > 2 days after symptom onset; *n* = 271) and early groups (remaining patients; *n* = 1802), and their clinical characteristics and mode of presentation were compared.

**Results:**

Age, sex, and laboratory findings including brain natriuretic peptide and serum creatinine levels were not significantly different between the two groups. Patients in the delayed group had greater chronic fluid retention and symptoms not associated with respiratory failure, whereas those in the early group were more likely to have acute respiratory distress, faster heart and respiration rates, and higher systolic blood pressure.

**Conclusions:**

More than one in ten patients with HF readmission delay seeking treatment > 2 days after symptom onset. Patients who delayed seeking treatment showed the phenotype of chronic fluid retention, whereas those who presented to the hospital earlier had the phenotype of acute respiratory failure.

## Background

In recent years, repeated emergent hospitalization for patients with acute heart failure (HF) has become a cause for significant medical and economic burden [1–4]. It is well recognized that educating patients about early signs of HF exacerbation and providing them with early medical intervention in ambulant settings are important to prevent emergent rehospitalization [5–8]. However, some patients who need readmission for HF delay asking for medical help after noticing their first symptoms, even though they have been made aware about the symptoms of HF exacerbation [2, 7, 9–14]. In a previous report, Shiraishi et al. [15] suggested that those who sought medical help earlier showed the phenotype of acute vascular failure, characterized by a transient volume shift from the peripheral veins to the pulmonary circulation, and those who delayed seeking treatment exhibited the phenotype of chronic fluid retention. Elucidating whether these characteristics are true in the setting of emergent readmission for HF is important, since theoretically, early medical intervention with diuresis may prevent readmission in patients with chronic fluid retention.

Our objectives were to describe the time interval between symptom onset and hospitalization in patients with HF readmission and to compare the clinical phenotypes of patients who delay seeking treatment before emergent readmission with those who presented to the hospital earlier.

## Methods

### Study design

Patient data were collected from the Tokyo Cardiovascular Care Unit (CCU) Network Registry. The Tokyo CCU Network Registry is a multicenter, prospective registry that includes consecutive patients requiring hospitalization due to acute cardiac disease via emergency medical services (EMS) [15–17]. A total of 72 hospitals in the Tokyo metropolitan area and the Tokyo Fire Department collaboratively participated in the registry. For this analysis, we retrospectively analyzed data from January 2014 to December 2015 in this registry. Each EMS unit collected information regarding the patient’s self-reported onset time of symptoms and the time when the patient required EMS. The data for patient’s self-reported symptoms, laboratory data, vital signs at admission, past medical history, primary etiology of the exacerbation, documentation of left ventricular ejection fraction (LVEF) via echocardiography, duration of hospitalization, and in-hospital mortality collected from medical records in each hospital were also registered. For this analysis, we included all registered symptoms in each patient. Information about the Tokyo CCU Network, data collection, and right of refusal to the enrollment to the registry were provided to patients via bulletin during hospitalization, and all personal data were blinded before the enrollment to the study.

### Eligibility criteria

**Fig. 1:**
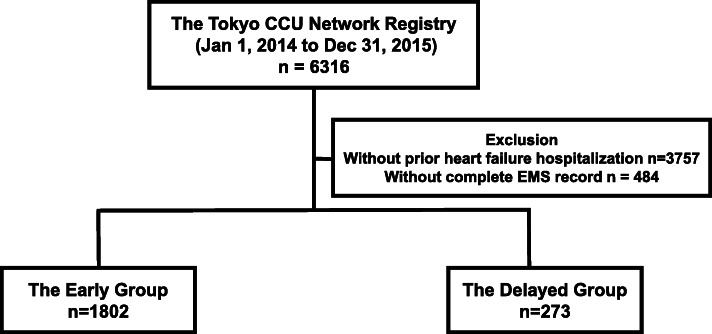
Study outline

A total of 6316 patients hospitalized for acute HF were transported to hospitals via EMS. The diagnosis of HF was made by the attending cardiologist in the respective hospitals. In the Tokyo CCU Network Registry, patients who presented with acute coronary syndrome were excluded from the HF registry. From these patients, we excluded patients without prior HF hospitalization (*n* = 3757), those who lack complete record from the EMS unit, and those without documentation of LVEF during hospitalization (*n* = 484) (Fig. 1).

### Early and delayed groups

We divided the patients into two groups according to the time duration between the onset of symptoms and the time when the patients called EMS. A total of 553 patients were unable to report the exact time of symptom onset and reported only the date of the symptom onset. For these patients, we were not able to calculate the precise time interval between the symptom onset and the time for EMS requirement. Therefore, we defined early group as those who called EMS in the same day or one day after the onset of the symptom. Theoretically, this definition was equal to those who called EMS within 48 h of symptom onset. Therefore, we defined these patients as early group (*n* = 1802) and the rest (those who required EMS 48 h after symptom onset) as delayed group (*n* = 271).

### Ethical approval

All procedures performed in studies involving human participants were in accordance with the ethical standards of the institutional and/or national research committee (Tokyo Saiseikai Central Hospital, approval number 29-39) and with the 1975 Declaration of Helsinki and its later amendments. As the study used anonymized data, informed consent was not required.

### Statistical analysis

Continuous variables were summarized with either mean ± standard deviation or median (25th and 75th percentiles) according to their distribution. Categorical variables were reported as percentage. The clinical characteristics of the early and the delayed groups were compared using Student’s *t* test, Pearson *χ*^2^ test, and Mann-Whitney *U* test as appropriate. All statistical analyses were performed using SPSS version 24 (IBM Corp., Armonk, NY, USA). A *P* value < 0.05 was considered statistically significant.

## Results

### Time interval between symptom onset and EMS requirement

Of the 2073 patients, 1802 were classified as the early group and 271 were classified as the delayed group. The distribution of the time interval is shown in Supplemental figure 1. One thousand one hundred sixty (64.3%) patients in the early group asked for EMS within 6 h after their symptom onset. Of the 553 patients with unknown time interval, 323 were classified as the early group and 230 were classified as the delayed group.

### Difference in patient characteristics between the early and delayed groups

Age and sex were not significantly different between the two groups. History of coronary artery disease and current dialysis were more prevalent in the early group, whereas history of atrial fibrillation was more prevalent in the delayed group. The proportion of hypertensive heart disease as the primary etiology of HF exacerbation was higher in the early group. Laboratory data including hemoglobin concentration, serum brain natriuretic peptide concentration, and serum creatinine concentration were comparable between the two groups. The distribution of LVEF and the prevalence of HF, with preserved (> 50%), mid-range (> 40 to ≤ 50%), and reduced LVEF (≤ 40%), were not significantly different between the two groups. Regarding the vital signs at admission, those in the early group showed faster heart rate, faster respiratory rate, and higher systolic blood pressure (Table 1).

**Table 1:** Patient characteristics

	Early (*n* = 1802)	Delayed (*n* = 271)	*P* value
Patient characteristics			
Age, year	78.5 ± 11.1	77.5 ± 13.1	0.23
Sex, female %	57.1	55.7	0.67
Past medical history			
Coronary artery disease, %	40.4	28.8	< 0.001
Hypertension, %	64.3	60.9	0.27
Diabetes mellitus, %	38.1	39.5	0.67
Dyslipidemia, %	28.9	26.9	0.51
Atrial fibrillation, %	35.8	45.1	0.003
Dialysis, %	5.8	2.6	0.03
Cerebrovascular disease, %	11.3	10.3	0.65
Primary etiology of heart failure			
Ischemic heart disease, %	28.2	24.4	0.19
Hypertensive heart disease, %	17.9	11.1	0.005
Arrhythmia, %	6.3	8.5	0.17
Cardiomyopathy, %	10.6	11.8	0.55
Valvular heart disease,%	20.3	23.2	0.27
Others, %	16.8	21.0	0.08
Laboratory data at admission			
Hb, mg/dl	11.6 ± 2.3	11.4 ± 2.2	0.09
BNP, pg/ml	800 (458 to 1475)	800 (428 to 1412)	0.14
Cr, mg/dl	1.26 (0.90 to 1.91)	1.26 (0.92 to 1.76)	0.14
Echocardiographic findings			
LVEF, %	42.0 ± 15.9	42.4 ± 16.8	0.74
Heart failure with preserved ejection fraction, %	33.1	36.2	0.27
Heart failure with mid-range ejection fraction, %	21.5	17.3	
Heart failure with reduced ejection fraction, %	45.4	46.5	
Vital signs at admission			
Heart rate, bpm	98.6 ± 26.3	91.8 ± 24.4	< 0.001
Respiration rate, times/m	23.2 ± 9.7	20.4 ± 8.4	< 0.001
Systolic blood pressure, mmHg	150.4 ± 37.4	138.3 ± 33.3	< 0.001
Percutaneous saturation of oxygen	93.3 ± 10.8	94.1 ± 12.9	0.25

*Hb* hemoglobin concentration, *BNP* plasma brain natriuretic peptide concentration, *Cr* serum creatinine concentration, *LVEF* left ventricular ejection fraction

### Symptoms at presentation

Nearly 90% of the patients in each group complained of shortness of breath. Peripheral edema and symptoms not associated with respiratory disorder, including abdominal discomfort, abdominal pain, back pain, and loss of consciousness, were more common in patients in the delayed group (Table 2).

**Table 2:** Presenting symptoms in each group

	Early (*n* = 1802)	Delayed (*n* = 271)	*P* value
Shortness of breath, %	88.7	86.0	0.20
Chest pain, %	12.3	9.2	0.15
Palpitation, %	3.6	3.7	0.95
Peripheral edema, %	2.9	8.9	< 0.001
Malaise, %	0.5	1.5	0.06
Symptoms not associated with respiration, %	14.0	26.9	< 0.001

Symptoms not associated with respiration included abdominal discomfort, abdominal pain, back pain, shock, and loss of consciousness.

### In-hospital mortality and duration of hospitalization

In-hospital mortality was comparable between the two groups (early group, 4.7%; delayed group, 6.3%). The median duration of hospitalization was longer in the delayed group than in the early group (delayed group, 21 days; early group, 15 days) (Table 3).

**Table 3:** In-hospital mortality and length of hospital stay

	Early (*n* = 1802)	Delayed (*n* = 271)	*P* value
In-hospital mortality, %	4.7	6.3	0.27
Duration of hospitalization, days	15 (10 to 25)	21 (12 to 33)	< 0.001

## Discussion

Patients in the early and delayed groups had distinct clinical characteristics; those in the delayed group showed the phenotype of chronic fluid retention and more frequently complained of symptoms not associated with respiratory distress, whereas those in the early group were more likely to have acute respiratory distress, higher blood pressure, and faster pulse and respiration rates. These findings suggest that examining the aforementioned phenotypes may be valuable in planning specific strategies to prevent rehospitalization for HF.

### Pathological differences between the early and delayed groups

Our results suggest that there are potential differences in pathophysiological characteristics between the early and late groups. Previous studies suggested that there are two pathophysiological causes of congestion in acute heart failure, fluid redistribution, and fluid retention. Fluid redistribution, also known as vascular failure, was characterized by rapidly progressive severe acute dyspnea and high blood pressure. The proposed mechanisms for this phenotype were acute vasoconstriction leading to secondary central volume shift and acute pulmonary edema, which were due to rapid activation of the sympathetic nervous system [18, 19]. These characteristics are well matched to the early group in our cohort, presenting acute respiratory distress, higher blood pressure, and faster pulse and respiration rates. A higher prevalence of hypertensive heart disease as the primary cause of the exacerbation in the early group also supports this notion. On the other hand, proposed characteristic findings of chronic fluid retention were milder and more slowly worsening symptoms, infrequent pulmonary edema, frequent jugular vein congestion, hepatomegaly, peripheral edema, signs of hepatic and renal dysfunction, and mental obtundation. The delayed group, who exhibited peripheral edema, abdominal symptoms, and lower blood pressure and respiration and pulse rates, was compatible with these characteristics. Paying attention to these pathophysiological differences may be important in planning early and patient-specific medical intervention. Regarding the SpO2 value, we did not observe a significant difference between the two groups. This finding was not in line with our hypothesis that those patients in the early group experienced more severe respiratory distress. One potential explanation for the lacking significant difference between these two groups was the effect of oxygen supplementation. Regrettably, we did not have the amount of oxygen supplemented in each patient; thus, the difference in the oxygen supplementation may have affected these results. Other important clinical factors, including renal function, plasma BNP, and left ventricular ejection fraction, did not differ significantly between two groups.

### Comparison to prior studies

There are several reports analyzing the delay in seeking treatment in HF. Our results can be distinguished from these previous reports as we specifically targeted patients with emergent HF rehospitalization, had a larger sample size and a multicenter design, and analyzed the pathophysiological differences between the early and delayed groups. Previous reports showed a delay interval between 2 h and 7 days, which were comparable to our results [2, 7, 11, 12]. Shiraishi et al. [15] reported an association between the time intervals between symptom onset and hospitalization and the difference in hemodynamic pathology, similar to that of our report. The fundamental difference between our results and those of Shiraishi et al. [15] was that we specifically analyzed patients needing readmission, since if the phenotype of the delayed group in these patients with readmission was also chronic fluid retention, the time delay may be utilized as potential therapeutic window.

Targeting specific populations is important for planning early and patient-specific intervention and education. However, narrowing the patient spectrum often reduces the statistical power of the analysis. The Tokyo CCU Network Registry has a relatively large sample size, thus providing an acceptable sample size for analyzing recurrent HF specifically. Most previous reports analyzed the association between patients’ characteristics including symptoms and delay in seeking treatment. In addition to this information, our results highlighted the pathophysiological differences between the early and delayed groups, which may lead to patient-specific and effective prevention of HF rehospitalization in both groups.

### Clinical implications

Our results may be valuable for patient education. More than one in 10 patients had > 2 days of delay after their onset of symptoms before seeking treatment. This delay could be used as a therapeutic window to prevent rehospitalization, since the clinical phenotype in the majority of these patients was chronic fluid retention, which theoretically can be modified with diuretics. Considering the enormous economical and medical burden of HF rehospitalization, educating these patients how to self-monitor the development of edema and how to contact the medical services without delay after the symptom onset may be valuable.

### Limitations

Our study does have certain limitations. First, the cut-off value of the time interval between the symptom onset and the EMS requirement as 48 h is relatively long for those who required emergent hospitalization. However, since approximately 25% of the patients were unable to provide the specific time of their symptom onset in our cohort, and only stated the date of their symptom onset, we were forced to group them into those with at least 48 h of delay and those with no more than 48 h of delay since we were not able to divide them with shorter cut-off value using only the date of the symptom onset. However, considering the need for therapeutic time window, this 2-day threshold may be reasonable. Second, we did not evaluate several factors reported to be associated with delay in seeking treatment including sociodemographic factors and access to medical services [11, 20]. This information is also important in planning the prevention strategy for HF readmission. Third, we were unable to analyze prior episodes of hospitalization for HF in each patient, including number and pathophysiological characteristics of prior hospitalizations for HF. In addition, we did not have the data regarding the duration between the prior hospitalization and the readmission. Future studies analyzing these characteristics may lead to further improvement in tailored education of patients with HF. Fourth, when analyzing the behavioral problems like appropriateness of treatment seeking, psychosomatic, and social factors are in negligible. To educate patients how to avoid rehospitalization, we should take into account the clinical phenotypes shown in this manuscript as well as psychosomatic and social factors. Lastly, in this analysis, we only analyzed patients who came to hospital via EMS. Since many patients with gradual recurrence of heart failure are thought to come to hospital without using EMS, this criterion may have limited the generalizability of our analysis. However, considering the facts that more than one in ten patients who came to hospital via EMS still took delay in seeking treatment and enormous economic and physical burden of EMS transportation, targeting patients who took time delay before calling for EMS may be of value.

## Conclusion

One in 10 patients with HF readmission had > 2 days of delay after symptom onset before seeking treatment. Patients who delay seeking treatment showed the phenotype of chronic fluid retention, in contrast with the acute respiratory failure phenotype seen in the early group. This information may be valuable in educating patients and in preventing rehospitalization for HF.

## Supplementary information

**Additional file 1: Supplemental Figure 1**. Distribution of time interval between symptom onset and the time patients asked for emergency medical services. *n* = 1173 (time interval < 6 h), 190 (time interval between 6 and 12 h), 83 (time interval between 12 and 24 h), 33 (time interval between 24 and 48 h), 323 (time interval < 48 h, the exact time interval not available), 41 (time interval over 48 h), 230 (time interval over 48 h, the exact time interval not available)

## Data Availability

The datasets generated and/or analyzed during the current study are not publicly available due that the authors do not have the right, but are available from the corresponding author on reasonable request.

## Abbreviations

CCU: Cardiovascular Care Unit
EMS: Emergency Medical Service
HF: Heart failure
LVEF: Left ventricular ejection fraction
