# Supplementary figures and images for: Delay in seeking treatment before emergent heart failure readmission and its association with clinical phenotype
Source: J Intensive Care. 2020 Aug 26;8:65. doi: 10.1186/s40560-020-00482-z (PMC7448509; doi:10.1186/s40560-020-00482-z)

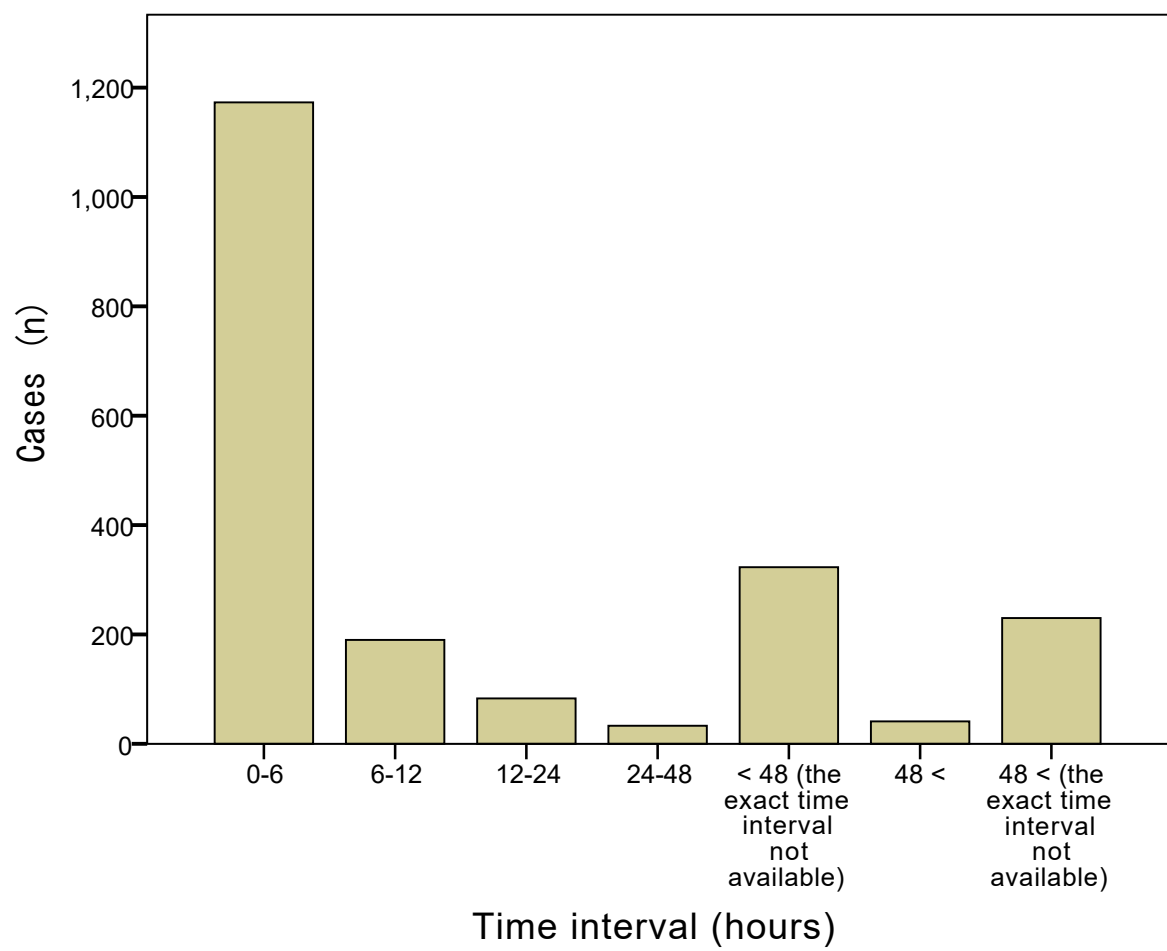

Supplement: Supplementary file 1 — Additional file 1: Supplemental Figure 1. Distribution of time interval between symptom onset and the time patients asked for emergency medical services. n = 1173 (time interval < 6 h), 190 (time interval between 6 and 12 h), 83 (time interval between 12 and 24 h), 33 (time interval between 24 and 48 h), 323 (time interval < 48 h, the exact time interval not available), 41 (time interval over 48 h), 230 (time interval over 48 h, the exact time interval not available) [file 40560_2020_482_MOESM1_ESM.pdf]
